# QuickStats

**Published:** 2014-03-21

**Authors:** FROM THE NATIONAL CENTER FOR HEALTH STATISTICS

**Figure:**
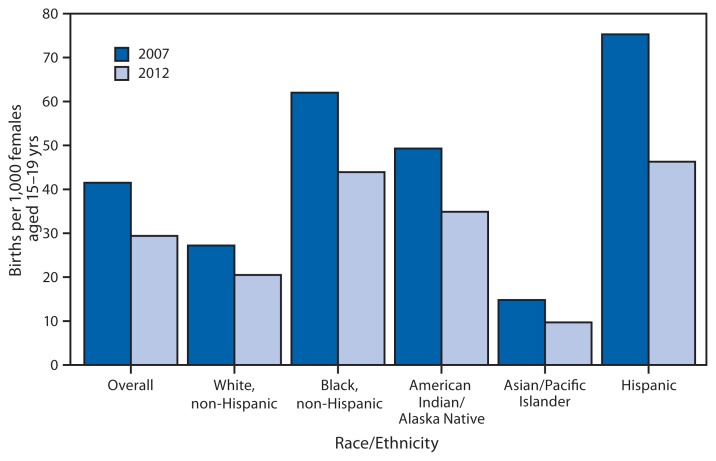
Birth Rates for Females Aged 15–19 Years, by Race/Ethnicity^*^ — National Vital Statistics System,^†^ United States, 2007 and 2012 ^*^ Persons categorized as American Indian/Alaska Native or Asian/Pacific Islander also might be Hispanic. ^†^U.S. residents only.

From 2007 to 2012, the birth rate for females aged 15–19 years in the United States overall declined by 29%, from 41.5 to 29.4 births per 1,000 females in that age group. Among racial/ethnic populations, declines ranged from 25% for non-Hispanic white females to 39% for Hispanics. Rates decreased 29% for non-Hispanic black females and American Indian/Alaska Natives and 34% for Asian/Pacific Islanders.

**Source:** Martin JA, Hamilton BE, Osterman JK, et al. Births: final data for 2012. Natl Vital Stat Rep 2013; 62(9). Available at http://www.cdc.gov/nchs/data/nvsr/nvsr62/nvsr62_09.pdf.

**Reported by:** Brady E. Hamilton, PhD, bhamilton@cdc.gov, 301-458-4653.

